# Xy-31 and Lat-1 regulate the induction of polysaccharide disintegrating genes by arabinose in *Neurospora crassa* OR74A

**DOI:** 10.3389/fmicb.2026.1880067

**Published:** 2026-08-27

**Authors:** Shriya Singh, Nidhi Adlakha

**Affiliations:** Synthetic Biology and Bioprocessing Lab, Regional Centre for Biotechnology, NCR-Biotech Cluster, Faridabad, Haryana, India

**Keywords:** arabinose, arabinose-proton symporter, cellulase, induction, sugar transporter

## Abstract

**Background:**

Arabinose has been identified as a potent inducer of the cellulase regulon in filamentous fungi. However, the precise molecular mechanisms that drive this induction remain largely unknown. This study aimed to elucidate the mechanisms underlying the arabinose-induced activation of cellulase and hemicellulase enzyme clusters in the model filamentous fungus *Neurospora crassa* OR74A.

**Results:**

Arabinose stimulates the secretion of cellulases and hemicellulases in *N. crassa*. While genome-wide scrutiny predicted several sugar transporters in *N. crassa*, expression studies under avicel and arabinan conditions indicated a notable upregulation of pentose transporters encoded by the lat-1 and xy-31 genes. The lat-1 and xy-31 deleted strains showed reduced arabinose uptake and diminished growth on arabinose media, highlighting their role in arabinose transport. Additionally, these transporter-deficient strains exhibited a significant decrease in endoglucanase and arabinase activity, which correlated with lower expression of cellulase-encoding transcripts, indicating the crucial roles of Lat-1 and Xy-31 in promoting the secretion of enzymes that degrade biomass. Consistent with these findings, overexpression of lat-1 and xy-31 in *N. crassa* resulted in a significant increase in cellulase secretion.

**Conclusion:**

We demonstrated that *N. crassa* has multiple pentose transporters. Furthermore, we validated that the Lat-1 and Xy-31 transporters play crucial roles in arabinose-mediated induction of polysaccharide-disintegrating enzymes in *N. crassa*. Both transporters are required for efficient arabinose uptake and for the subsequent arabinose-dependent induction of polysaccharide-disintegrating enzymes. Collectively, these findings significantly advance the molecular understanding of fungal hemicellulose utilization and identify key transporters that represent promising targets for enhancing fungal biomass bioconversion.

## Background

The escalating demand for non-renewable energy sources has underscored the need for alternative fuels. Second-generation biofuel presents a compelling alternative to traditional fuels, as it depends on abundant and renewable lignocellulose as feedstock (Mujtaba et al., 2023). To effectively utilize this abundant feedstock, biomass must be saccharified into monomeric sugars. A cocktail of cellulolytic enzymes is required to hydrolyze complex lignocellulose into fermentable monomeric sugars (Nogueira et al., 2020; Adsul et al., 2020). To address this, researchers have turned their attention to fungi as potential sources of cost-effective cellulolytic enzymes.

Cellulases are adaptive enzymes that are specifically induced by polysaccharide substrates in filamentous fungi (Yan et al., 2021). Recent studies have shown that saccharide inducers such as cellobiose, lactose, and sophorose can also stimulate the secretion of polysaccharide-degrading enzymes (Amore et al., 2013). These soluble inducers employ sugar transporters to mediate cellulase induction, either by facilitating their entry into the cell or by sensing inducers (Wang et al., 2022). For instance, CDT-1/2 are the first reported cellobiose transporters in *N. crassa,* and their deletion led to a marked decrease in the expression of genes involved in plant cell wall deconstruction (Cai et al., 2014). Furthermore, Znameroski et al. (2014) confirmed that this transporter plays an indispensable role in signaling cellulase induction by crystalline cellulose, validating its role as a transceptor (Znameroski et al., 2014). Similarly, HGT-1/2 and GLT-1/2 are high- and low-affinity glucose transporters that play a pivotal role in glucose-mediated catabolite repression of cellulase enzymes by modulating the cyclic AMP–PKA pathway (Wang B. et al., 2017). Although hexose-based inducers have been studied in detail, the role of arabinose in cellulase and hemicellulase induction has not yet been elucidated. This study aimed to identify the specific transporters involved in the arabinose-mediated induction of cellulase and hemicellulase genes in *N. crassa* and to elucidate the underlying molecular mechanisms.

Here, we have shown that five-carbon arabinose elicits induction of cellulases and hemicellulases in the model strain *N. crassa* OR74A. *In silico* predictions, supported by RT-qPCR, identified pentose transporter candidates associated with biomass-degrading enzyme regulation. We observed the involvement of genes encoding arabinose proton symporters (Lat-1) and quinate permeases (Xy-31) in arabinose-driven induction. Lat-1 and Xy-31 are arabinose transporters in *N. crassa* that play crucial roles in arabinose uptake and cellulase induction. The deletion of these genes reduces arabinose utilization and enzyme activity, whereas their overexpression enhances cellulase and arabinase production, suggesting their importance in regulating the secretion of plant biomass-degrading enzymes.

## Methods

### Fungal strains, media, and culture conditions

All single-deletion strains of *N. crassa* were obtained from the Fungal Genetic Stock Center (FGSC, Manhattan, KS, USA) (Colot et al., 2006). The strains included wild-type (FGSC 2489) and the knockouts, namely, ΔNCU02188 (FGSC 16052), ΔNCU05897 (FGSC 13716), ΔNCU06138 (FGSC 13566), ΔNCU08152 (FGSC 22811), ΔNCU09287 (FGSC 18265), ΔNCU10021 (FGSC 22818), ΔNCU00801 (FGSC 16575), ΔNCU01132 (FGSC 17613), ΔNCU05853 (FGSC 13771), and ΔNCU00988 (FGSC 16537). The wild-type strain and its mutants were maintained on potato dextrose agar (PDA). For the enzyme assays and RT-qPCR studies, the strains were cultivated in Vogel’s medium N (Vogel, 1956) supplemented with 2% arabinan (Megazyme) or 2% avicel (Merck), in separate experiments. A sorbose-containing medium (FGS) was employed to facilitate ascospore germination and to isolate transformant colonies (Davis and de Serres, 1970). The culture was incubated at 28 °C for 6 days, with rotary shaking at 180 rpm, unless otherwise defined. The clarified culture supernatant was used for the enzyme analysis, and the mycelial pellet was harvested for RNA extraction.

### *In silico* analysis of sugar transporters

The genomic sequence of *Neurospora crassa* OR74A v2.0 was analyzed for the presence of sugar transporters. This was obtained using the Mycocosm server (Grigoriev et al., 2014) in conjunction with previously reported sugar transporters. Sugar transporters (STs) were screened based on their transmembrane domains and signal peptides. The transmembrane domains were predicted using DeepTMHMM 1.0 (Hallgren et al., 2022), which analyzes protein sequences to identify membrane-spanning regions. Signal peptides were predicted using SignalP-5.0 (Nielsen et al., 2019). The subcellular localization of sugar transporter proteins were predicted using WoLF PSORT (Horton et al., 2007). Additionally, the sugar transporters domain (PF00083) were identified by searching the InterPro database (Blum et al., 2025), which classifies protein families and provides functional annotations based on conserved domain signatures. The transcriptome database was compiled based on previous studies by Coradetti et al. (GSE35227) (Coradetti et al., 2012), Xiong et al. (GSE92848) (Xiong et al., 2017), Znameroski et al. (GSE36719) (Znameroski et al., 2012), and Wang et al. (GSE60986) (Wang et al., 2015).

### Real-time quantitative PCR (RT-qPCR)

The wild-type *Neurospora* spores (1*10^7^ spores/mL) were inoculated in 50 mL of 1 × Vogel’s media containing 2% glucose and incubated at 28 °C under rotary shaking at 180 rpm. The mycelia were harvested after 18 h using centrifugation and transferred to fresh induction media containing a 2% carbon source (glucose, arabinan, or avicel). The mixture was incubated for an additional 7 h. The mycelia were then collected using a mira cloth. RNA was extracted using the RNeasy Mini Kit (Qiagen) following the manufacturer’s protocol. Residual genomic DNA was eliminated by treating samples with RNase-free DNase I (Qiagen). The RNA integrity and concentration were analyzed using agarose gel electrophoresis and a NanoDrop spectrophotometer, respectively. For cDNA synthesis, 4 μg of total RNA was reverse transcribed using the High-Capacity cDNA Reverse Transcription Kit (Thermo Fisher Scientific) under the following thermal conditions: 25 °C for 10 min, 37 °C for 120 min, and 85 °C for 5 min. Quantitative PCR was performed using the Quant Studio 6 Flex System in a 15 μL reaction mixture containing 7.5 μL of 2 × TB Green Premix Ex Taq (Tli RNaseH Plus), 0.3 μL of 50 × ROX reference dye II, 1 μL each of 10 μM forward and reverse primers, 1 μL of cDNA template, and nuclease-free water. The amplification protocol consisted of an initial denaturation at 95 °C for 30 s, followed by 40 cycles of 95 °C for 5 s, 55 °C for 10 s, and 60 °C for 20 s. The primers used for RT-qPCR analysis are presented in Supplementary Table S1. Primer annealing temperatures were optimized using gradient PCR.

Following amplification, melt-curve analysis was performed to verify the specificity of the PCR products and to confirm the absence of primer-dimer formation. Relative gene expression levels were determined using the 2^-ΔΔCt method (Livak and Schmittgen, 2001). *β*-actin served as the internal reference gene because of its stable expression under the experimental conditions. Error bars represent standard deviations derived from three independent experiments. The negative control contained DEPC-treated water instead of the RNA sample.

### Biochemical analysis

The biomass-degrading capability of *Neurospora* sp. was analyzed using various biochemical assays. Endoglucanase/Xylanase assay. Briefly, 150 μL of the supernatant was mixed with an equal volume of 1% CMC/xylan solution in 100 mM citrate buffer (pH 4.8). The reaction mixture was incubated at 50 °C for 30 min (Aggarwal et al., 2023; Singh et al., 2025). The amount of reducing sugars released was measured using the DNSA (3,5-dinitrosalicylic acid) method at 540 nm (McCleary and McGeough, 2015). Standard curves were generated with glucose and xylose (0.1–0.8 mg/mL) to estimate cellulose and xylan degradation, respectively, facilitating the absolute quantification of reducing sugars. An enzyme blank with assay buffer, substrate, and DNS reagent, as well as a substrate blank with assay buffer, DNS reagent, and supernatant (enzyme), were utilized to correct the absorbance of the test samples. Similarly, arabinase activity in the culture supernatant was determined by measuring the reducing sugars produced by arabinan hydrolysis. Briefly, 150 μL of the supernatant was mixed with an equal volume of 1% arabinan solution in 100 mM citrate buffer (pH 4.8). The reaction mixture was incubated at 50 °C for 30 min (Sakamoto and Thibault, 2001). The amount of reducing sugars released was measured using the DNSA method at 540 nm. A standard curve was prepared using arabinose standards (0.1–0.9 mg/mL) to quantify arabinose in the test samples. One unit of enzyme activity was defined as the amount of enzyme required to release 1 μmol of reducing sugar per minute with a given substrate (Adlakha et al., 2011). Appropriate substrate and enzyme blanks were used to correct the absorbance measurements of both standards and test samples.

### Sugar uptake assay

Equal spore counts (1*10^7^ spores/mL) of the wild type, Δlat-1, and Δxy-31 strains were inoculated separately in 1 × Vogel’s media containing 33.33 mM arabinose [0.5% (w/v) arabinose]. The culture mixture was collected every 24 h for 7 days. Culture supernatants were filtered using a 0.22-μm membrane filter before HPLC analysis. A 10 μL sample was injected into the Rezex-ROA Organic acids column, and 0.001 N H_2_SO4 was used as the mobile phase at a flow rate of 0.6 mL/min and a column temperature of 60 °C using an Agilent 1,260 Infinity II HPLC system equipped with a refractive index detector (Jeong et al., 2020). To quantify residual arabinose in the test samples, a standard curve was made using arabinose standards ranging from 2 to 7 mg/mL. Arabinose was identified by its retention time (11.009 min). Three independent biological replicates were tested. Vogel’s medium with 33.33 mM L-arabinose was used as the analytical blank.

### Bright field microscope analysis

Bright-field microscopy images of the wild type, Δlat-1, and Δxy-31 strains were obtained using a binocular compound microscope (LMI, London, UK) equipped with a 40x objective lens. For staining, mycelial samples from the wild-type, Δlat-1, and Δxy31 strains were directly collected from PDA plates. The samples were then incubated for approximately 1 min in a drop of lacto-phenol blue, rinsed with Milli-Q water (MQ), air-dried, and subsequently used for microscopic examinations (Shamly et al., 2014).

### Colony diameter measurement and sporulation assay

The conidia (2*10^7^ spores) of the wild-type *N. crassa*, Δlat-1, and Δxy31 strains were separately inoculated in Vogel’s minimal (VM) media plates supplemented with 2% glucose, xylose, arabinose, lactose, avicel, and a combination of 1% avicel and 1% wheat bran. The plates were incubated at 28 °C for 19 h. Colony diameters were measured at designated time points (Data not shown), and representative images were captured. Additionally, spores were harvested from VM plates supplemented with 2% glucose. Spore counts were determined using a hemocytometer under a bright-field microscope with a 40x objective (Wang et al., 2023). Error bars represent standard deviations derived from three independent experiments.

### Transformation

For transformation, 40 μL of conidial suspension (approximately 1*10^8^ conidia) was mixed with 300 ng of plasmid DNA in a sterile microcentrifuge tube. The suspension was gently mixed and kept on ice for at least 5 min. The conidia–DNA mixture was transferred into a pre-chilled 0.2 cm electroporation cuvette (Bio-Rad). Electroporation was carried out at a voltage gradient of 7.5 kV cm^−1^ (1.5 kV in a 0.2 cm cuvette), with a capacitance of 25 μF and resistance of 600 *Ω*. Immediately after electroporation, the mixture was resuspended in 1 mL of 1 M sorbitol and mixed gently by pipetting. The electroporated conidia–DNA mixture was mixed with molten FGS top agar and poured onto solidified FGS bottom agar supplemented with glufosinate ammonium (300 μg/mL) (Laxmi and Tamuli, 2017). The plates were subsequently incubated at 30 °C. Successful transformants were confirmed by RT-qPCR analysis.

### Overexpression of lat-1 and xy-31

For lat-1 and xy-31 overexpression, the coding genes were cloned under the Ptcu-1 promoter, and the two constructs pRS426-Ptcu-1-lat-1 and pRS426-Ptcu-1-xy-31 were independently transformed into the wild-type strain (FGSC 2489) using electroporation to generate the overexpression strains. For biochemical analyses, the wild-type and overexpression strains were pre-cultured in Vogel’s medium supplemented with CuSO₄ (50 μM) for 24 h. Subsequently, cultures were transferred to inducing conditions containing VM supplemented with 2% arabinan (along with 200 μM bathocuproine disulfonic acid, BCS) and incubated for an additional 72 h (Lamb et al., 2013). The culture supernatants were collected by filtration and used for enzymatic assays.

## Results

### Arabinose induces the secretion of biomass-degrading enzymes

Previously, we have demonstrated that simple sugars such as cellobiose and gentiobiose can augment cellulase production in filamentous fungi by more than threefold (Aggarwal et al., 2023). Other six-carbon sugars, such as lactose and sophorose, have also been shown to enhance cellulase secretion in *T. reesei* (Akel et al., 2009; Havukainen et al., 2021), highlighting the importance of inducers in cellulase secretion. Arabinose, a pentose sugar, has also been proposed as an inducer of hemicellulolytic enzymes in *T. ressei* (Akel et al., 2009). Nevertheless, the underlying mechanisms for induction remain unelucidated.

This study was designed to investigate the arabinose-mediated induction using *N. crassa* as a model organism. For this purpose, *Neurospora* sp. was cultivated in media containing 0, 1.3, 6.6, 66, and 133 mM arabinose as the sole carbon source for 3 days at 28 °C. Glucose at comparable concentrations was used as a negative control in the experiment. The samples were collected every 24 h to estimate biomass and cellulase secretion. Since *N. crassa* exhibits a lower rate of arabinose utilization and shows complete utilization on the third day, 72 hpi was selected for studying the induction mechanism in the fungi.

**Figure 1:**
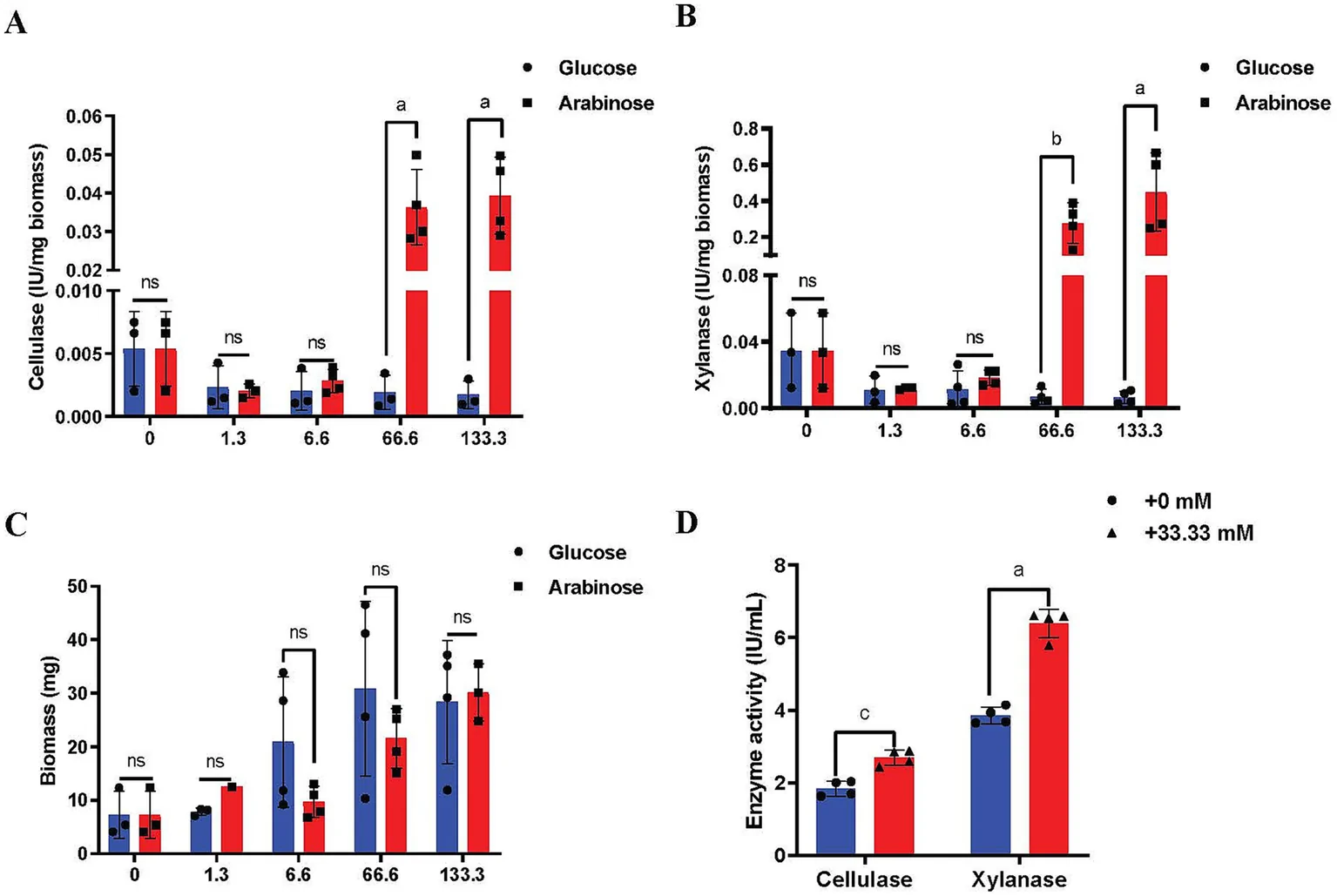
Arabinose induces the secretion of biomass-degrading enzymes. The culture supernatant of *N. crassa* grown in various concentrations of glucose or arabinose (0, 1.3, 6.6, 66.6, and 133.3 mM), and cellulase **(A)** and xylanase **(B)** activity using the standard DNSA method. **(C)** Bar graphs represent total fungal biomass obtained when *N. crassa* was cultured separately in different concentrations of arabinose and glucose as the sole carbon source. **(D)** The effect of arabinose supplementation on cellulase induction was analyzed by adding 33.33 mM arabinose to avicel media, and the culture supernatant was analyzed for an increase in cellulase and hemicellulose activity. Bars represent the mean ± SD of three independent biological replicates. Statistical significance between glucose (control) and arabinose at each corresponding concentration was determined using a two-way ANOVA followed by Šídák’s multiple-comparison test. Different letters indicate levels of statistical significance: a, *p* < 0.0001; b, *p* < 0.001; c, *p* < 0.01; d, *p* < 0.05; ns, not significant.

*N. crassa* showed a proportional enhancement in enzyme secretion with increasing arabinose concentration, and saturation was observed at 66 mM concentration. However, glucose significantly repressed the secretion of polysaccharide-disintegrating enzymes (Figures 1A,B). Interestingly, *N. crassa* showed similar total biomass production in arabinose and glucose (Figure 1C), suggesting that increased cellulase secretion in fungi is not associated with growth but instead involves an alternate signaling mechanism.

Additionally, supplementing arabinose into avicel media resulted in a > 1.5-fold increase in cellulase and hemicellulase activity in *N. crassa* (Figure 1D), validating the role of arabinose as an inducer. Overall, enzyme secretion in *N. crassa* was linearly correlated with increasing arabinose concentration, suggesting that arabinose plays a critical role in activating the cellulase enzyme operon. The potential mechanism could involve arabinose-specific transporters, as observed in other fungi (Havukainen et al., 2021), in which sugar uptake triggers a cascade of gene regulation leading to enzyme secretion.

**Figure 2:**
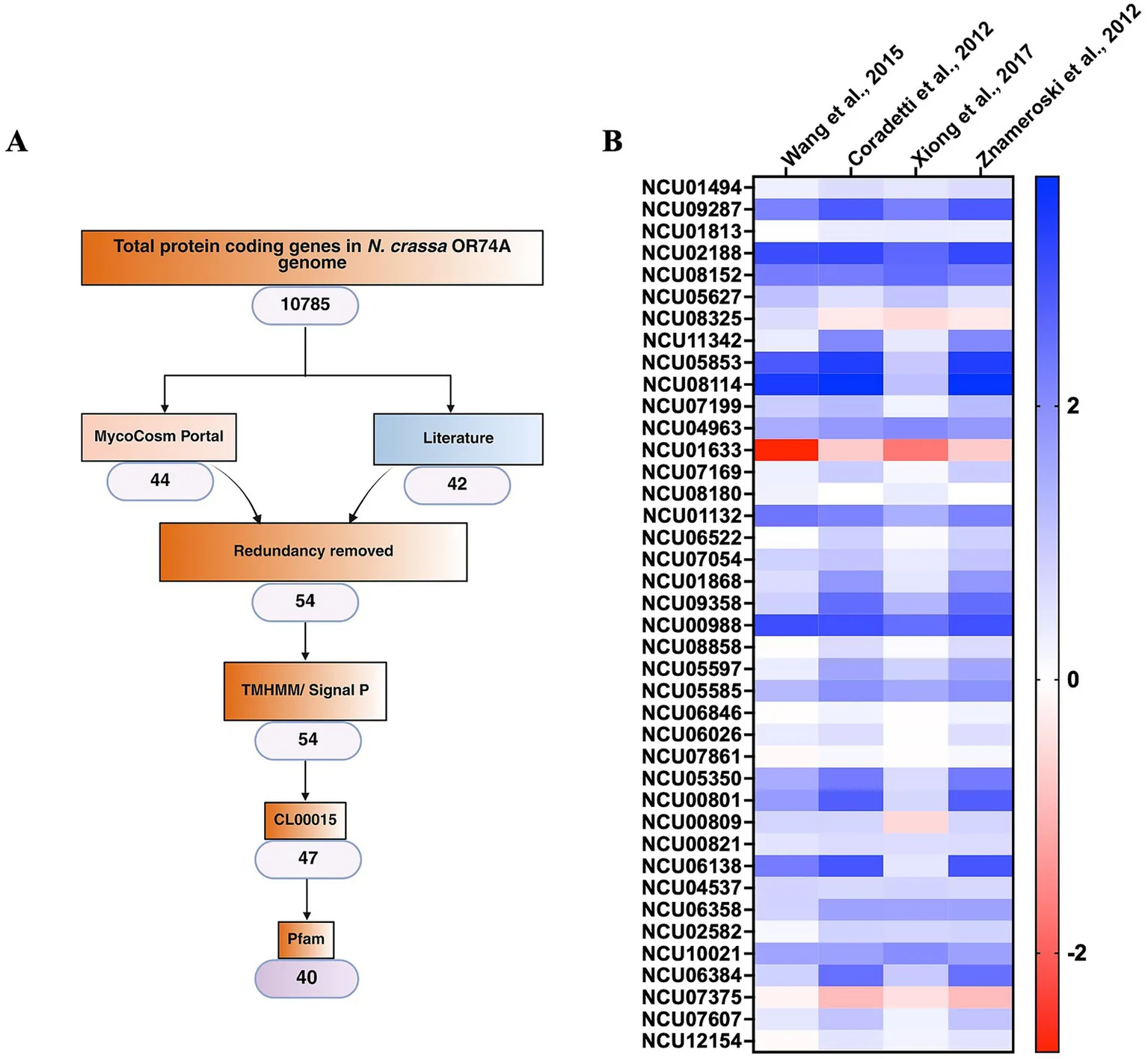
*In silico* identification of arabinose transporter. **(A)** The total number of protein-coding genes in *N. crassa* was determined through literature mining, domain analysis, and the MycoCosm portal. The membrane localisation of transporters was ascertained by TMHMM or WoLF PSPOT web server. **(B)** The expression of all the STs in the presence of the polysaccharide substrate was analyzed using the FungiDB server, based on transcriptomic datasets from Coradetti et al. (GSE35227), Xiong et al. (GSE92848), Znameroski et al. (GSE36719), and Wang et al. (GSE60986), which identified 10 upregulated sugar transporters.

### Identification and functional characterization of candidate arabinose transporters

Cell surface proteins are the first point of contact for relaying signaling messages. Znameroski et al. validated the role of CDT-1 and CDT-2 transporters in sensing and relaying the signaling message to the cellulase secretion machinery in fungi (Znameroski et al., 2014). In contrast, knockout of STP1 has been shown to increase cellulase activity in filamentous fungi (Zhang et al., 2015), suggesting the critical role of transporters in modulating the cellulase secretion pathway.

To gain a deeper understanding of how arabinose induces enzyme secretion in *N. crassa*, we first conducted an in-depth analysis of arabinose transporters in *N. crassa.* A comprehensive analysis of 10,785 protein-coding genes in the *N. crassa* OR74A genome was conducted using a reverse BLAST search and the Mycocosm portal, identifying 40 sugar transporters in this fungus (Figure 2A). The molecular weights of these transporters ranged from 66 to 89 kDa. The WoLF PSORT Prediction and TMHMM tools were used to determine the subcellular localization of sugar transporters, revealing that most are membrane-bound, suggesting a potential role in interacting with extracellular saccharides (Supplementary Table S2). To further characterize sugar transporters for their potential roles in arabinose uptake and cellulase induction, we conducted *in silico* analyses, including transcriptomic data mining and motif analysis.

Expression analysis of sugar transporters in the presence of polysaccharide substrates was analyzed using the FungiDB database (Figure 2B). Progressive multiple sequence alignment of the 10 upregulated sugar transporters revealed that the majority contain the conserved hexose-recognition motif PESPR, whereas a subset, including NCU06138 (Xy31), NCU10021, NCU02188 (Lat-1), and NCU08152, harbors the pentose-specific motif G-G/FXXX-G (Supplementary Figure S1), (Rani et al., 2016; Wang C. et al., 2017). Having identified potential sugar transporters through bioinformatic analyses, we investigated their functional relevance in enzyme induction using expression profiling and gene knockouts.

**Figure 3:**
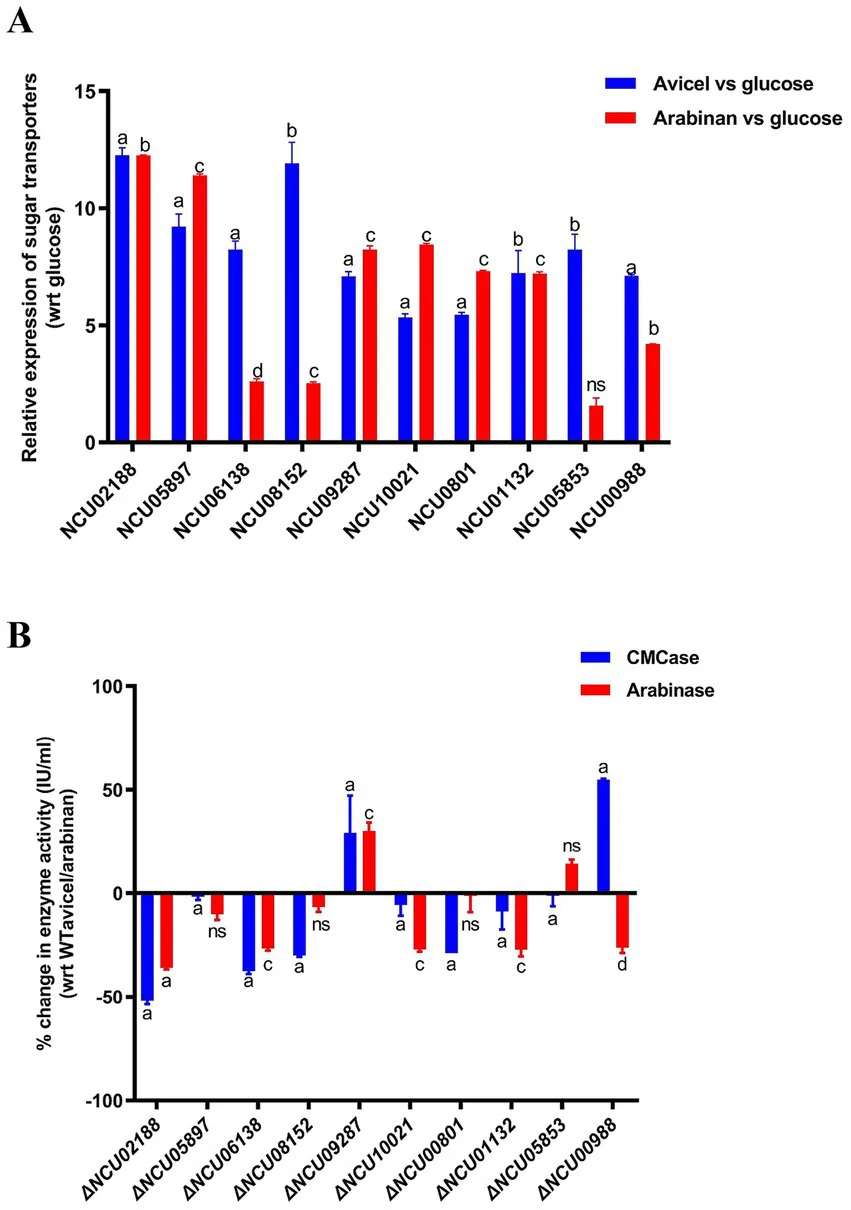
Deletion of lat-1 and xy-31 shows repression in cellulase activity. **(A)** The wild-type *N. crassa* was grown in the presence of avicel and arabinan, and RNA was extracted from the mycelial pellet after 24 h. RT-qPCR was performed to ascertain the expression of predicted upregulated sugar transporters. **(B)** The single-gene deletion mutants of these STs were acquired from FGSC and cultivated in polysaccharide medium. The culture supernatant was harvested after 5 days and analyzed for the percentage change in cellulase and arabinase activity relative to wild-type *N. crassa*. Bars represent the mean ± SD of three independent biological replicates. Statistical significance was determined using two-way ANOVA followed by Sidak’s multiple comparisons test. Different letters indicate levels of statistical significance: a, *p* < 0.0001; b, *p* < 0.001; c, *p* < 0.01; d, *p* < 0.05; ns, not significant.

The real-time expression analysis further revealed that all sugar transporters—predicted to be upregulated in the presence of polysaccharide substrate showed increased expression under both avicel and arabinan (Figure 3A). The knockout strains of selected transporters were procured from the FGSC and cultivated in Vogel’s media containing avicel and arabinan, separately. The role of sugar transporters was investigated by measuring enzyme activity in the culture supernatants of the corresponding deletion strains. Among the sugar transporters studied, deletion of the lat-1 and xy-31 genes significantly reduced enzyme activity toward both avicel and arabinan (Figure 3B), suggesting a potential role for the pentose transporters Lat-1 and Xy-31 in enzyme secretion.

### Lat-1 and xy-31 are involved in inducing cellulase activity

**Figure 4:**
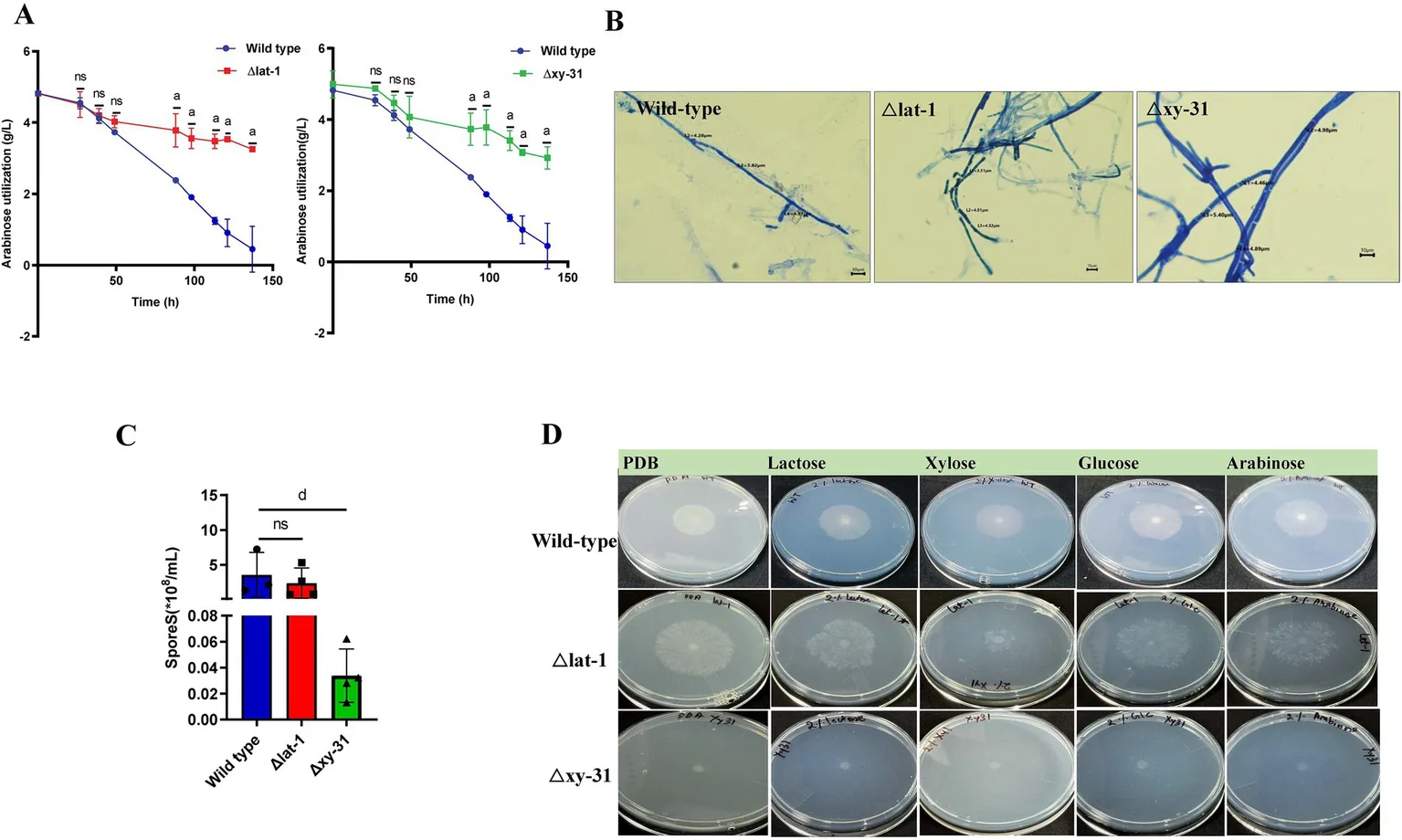
Arabinose utilization, growth, morphology, and sporulation in lat-1 and xy-31 knockout strains. **(A)** The wild type and mutant strains were cultivated in 33.33 mM arabinose, and the residual arabinose in the culture supernatant was measured using HPLC analysis. **(B)** The filamentous growth of pre-stained wild-type and mutant fungi was captured at 40X, which depicts similar mycelial morphology. **(C)** The sporulation of wild type and mutant strains. **(D)** An equal amount of spores was placed at the center of the plate, and growth was measured after 19 h. Bars represent the mean ± *SD* of three independent biological replicates. Statistical significance was determined using two-way ANOVA followed by Sidak’s multiple comparisons test. Different letters indicate levels of statistical significance: a, *p* < 0.0001; b, *p* < 0.001; c, *p* < 0.01; d, *p* < 0.05; ns, not significant.

Lat-1 has been characterized as an arabinose-proton symporter, facilitating the transport of arabinose molecules across the cell membrane of *N. crassa* (Li et al., 2015), whereas Xy-31 is classified within the major facilitator superfamily (MFS) and participates in arabinose and glucose uptake (Glass et al., 2014). In concordance, we observed reduced arabinose utilization in the lat-1 and xy-31 knockout strains (Figure 4A), confirming their role in arabinose transport. Although the deletion of lat-1 and xy-31 did not affect morphology in *N. crassa* (Figure 4B), a reduction in fungal sporulation was observed in the xy-31-deleted strain (Figure 4C). It was also clear from the plate-based growth assay, wherein equal number of spores were spotted at the center of the agar plate, and the xy-31 deleted strains exhibited a notable decrease in growth on lactose, glucose, xylose and arabinose media, likely due to reduced rate of substrate utilization in the knockout strains (Figure 4D). However, when an equivalent amount of mycelia was used to inoculate Vogel’s medium containing arabinan, the deletion strain did not exhibit any growth defects (Supplementary Figure S2). Overall, the data indicated that Lat-1 and Xy-31 play a role in the uptake of arabinose and other simple saccharides, but do not affect growth in complex cellulose media.

**Figure 5:**
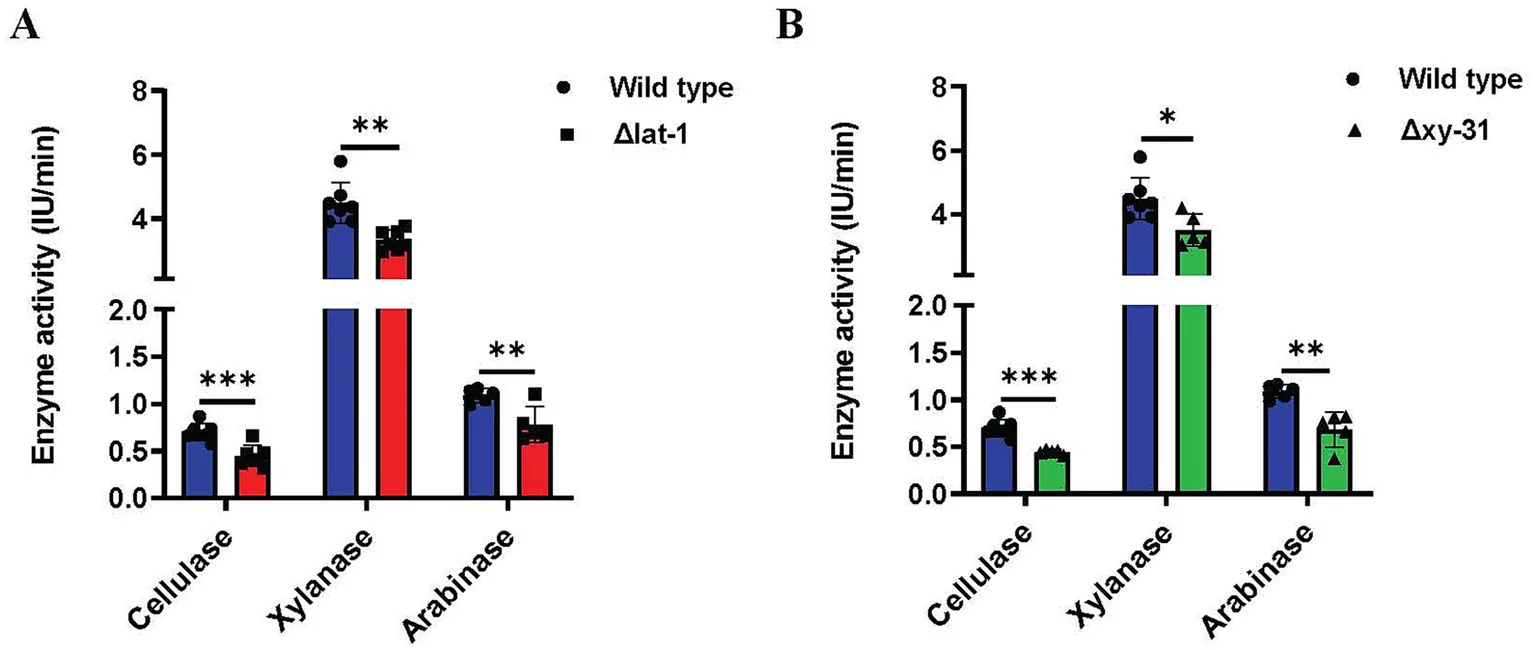
Effect of transporter gene deletion on enzyme induction. **(A)** The cellulase, xylanase, and arabinase activity was significantly reduced in △lat-1 **(A)** and △xy-31 **(B)** strains. The blue bar indicates the wild-type strain, the red and green bars indicate △lat-1 and △xy-31, respectively. Bars represent the mean ± *SD* of three independent biological replicates. Statistical significance was determined using multiple unpaired *t*-tests (one per row). Asterisks indicate significant differences between the wild type and mutant strains for each enzyme (*p < 0.05, **p < 0.01, ***p < 0.001, ****p < 0.0001; ns, not significant).

We assessed enzyme activity in the lat-1 and xy-31 deleted strains grown on arabinan, a complex arabinose-based polysaccharide, as the carbon source. The results indicated a substantial decline in both cellulase and hemicellulase activities in the deleted strains (Figures 5A,B). These results led us to believe that the Lat-1 and Xy-31 transporters may play a vital role in the arabinose-mediated induction of cellulase and hemicellulose secretion. These genes may be involved in signaling pathways that detect arabinan and trigger the production of cellulases and hemicellulases. Further investigation into the specific mechanisms by which these genes influence enzyme activity could provide valuable insights into the regulation of plant cell wall degradation. Consistent with these findings, the expression of cellulase- and hemicellulase-encoding genes in the lat-1 and xy-31 deletion strains was consistent with the biochemical assay results. Specifically, cbh-2 (NCU09680), endo-beta-1,4-xylanase (NCU08189), and arabinosidase (NCU05965) exhibited the downregulation in transporter mutants (Supplementary Figure S3), suggesting their role in cellulase induction.

**Figure 6:**
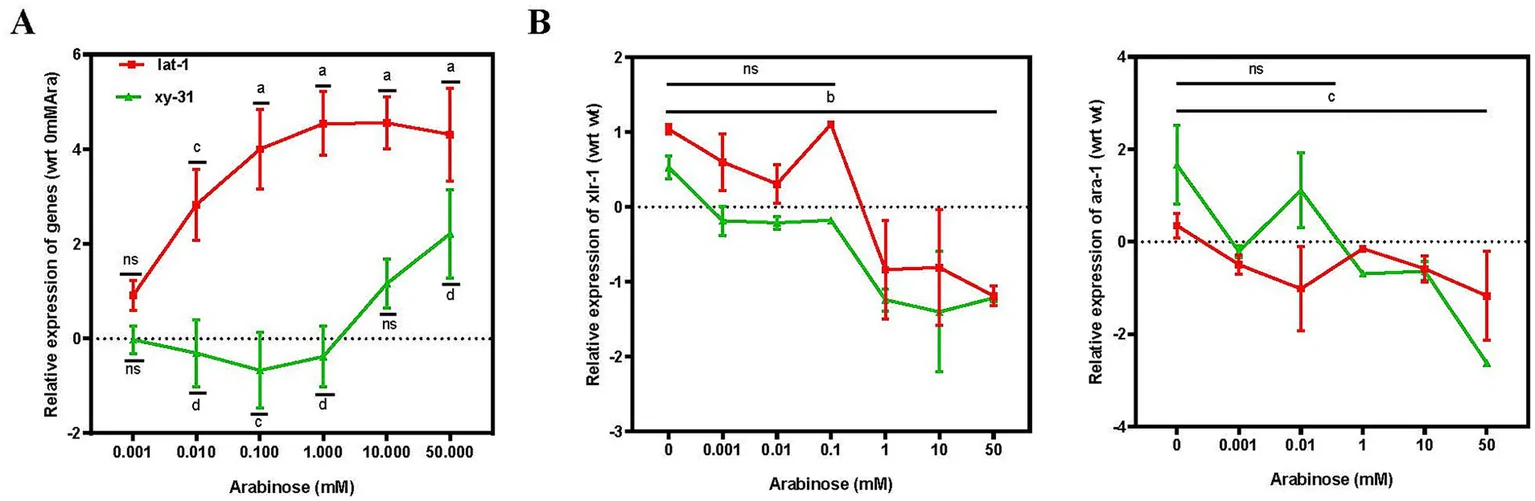
Crosstalk between transporters. **(A)** Expression of lat-1 and xy-31 was analyzed in wild-type *N. crassa* at various concentrations of arabinose. **(B)** Expression of xlr-1 and ara-1 was analyzed in wild-type and deletion mutants at various arabinose concentrations. The data for lat-1 are represented by dark circles, while those for xy-31 are shown with dark squares. Bars represent the mean ± SD of three independent biological replicates. Statistical significance was determined using a two-way ANOVA followed by Šídák’s multiple-comparison test. Different letters indicate levels of statistical significance: a, *p* < 0.0001; b, *p* < 0.001; c, *p* < 0.01; d, *p* < 0.05; ns, not significant.

To understand the crosstalk of Xy-31 and the Lat-1 transporter in cellulase induction, we cultivated *N. crassa* OR74A, Δlat-1, and Δxy-31 in arabinose at concentrations ranging from 0.001 mM to 50 mM for 24 h. First, we analyzed the expression of the lat-1 and xy-31 genes in response to extracellular arabinose in the wild-type strain of *N. crassa*. The lat-1 gene exhibited basal expression at lower arabinose concentrations, and its expression increased in a dose-dependent manner as arabinose concentration rose. Conversely, the xy-31 gene expression was reduced at lower arabinose levels but was upregulated when the arabinose concentration was higher (Figure 6A). These results suggest the possibility of crosstalk between lat-1 and xy-31 expression in facilitating the induction process.

Furthermore, we examined the involvement of candidate transcriptional activators, including Xlr-1 and Ara-1, in arabinose-mediated induction (Furukawa et al., 2009). Stricker et al. (2006) demonstrated that in *Trichoderma* sp., XYR1 functions as the master regulator of cellulase and xylanase gene expression, as deletion of XYR1/XlnR/XLR-1 led to a marked decrease in enzyme activity (Stricker et al., 2006). The deletion of ara-1 led to a significant reduction in the expression of arabinanolytic enzyme-encoding genes in *Myceliophthora thermophila* (Gu et al., 2023). As a result, we investigated the role of the transporter in regulating the expression of transcription factors, such as Xlr-1 and Ara-1, in *N. crassa*. Our findings revealed that the transporter’s absence led to a significant decrease in the expression levels of these transcription factors. This decline was consistently observed across a range of arabinose concentrations (Figure 6B). Overall, it appears that the Xlr-1 and Ara-1 are potent transcription factors involved in Lat-1 and Xy-31-mediated induction in *N. crassa.*

### Overexpression of lat-1 and xy-31 in *Neurospora crassa*

**Figure 7:**
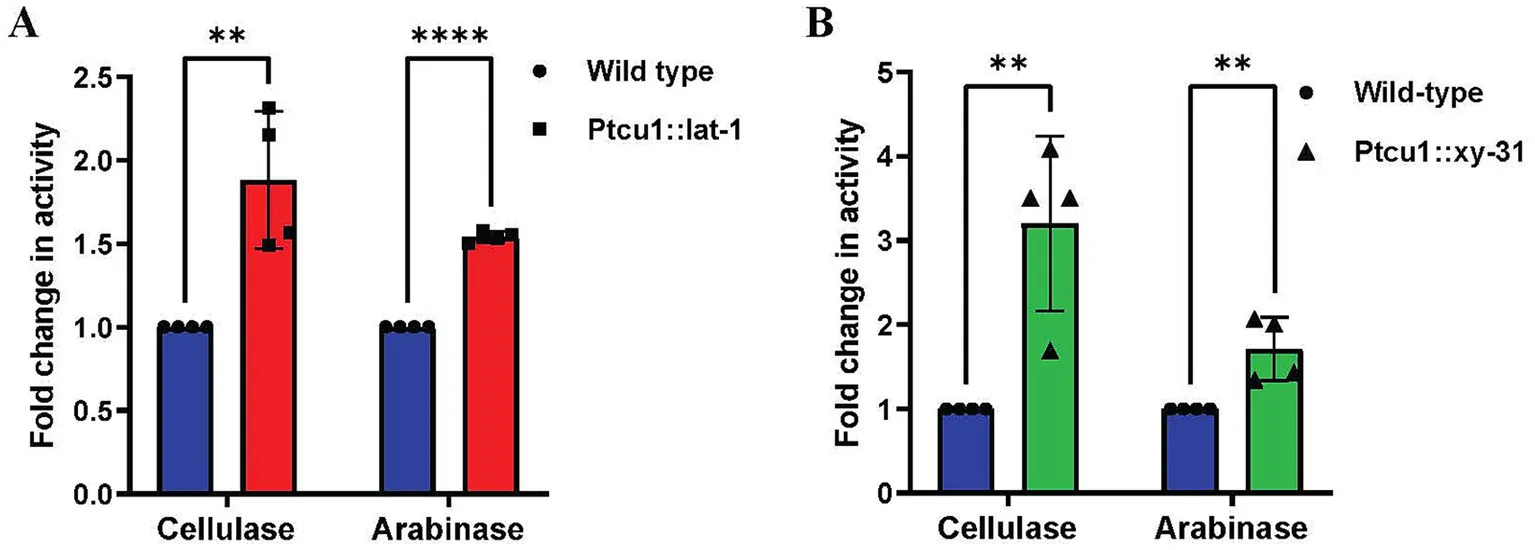
Overexpression of transporters. Overexpression of lat-1 **(A)** and xy-31 **(B)** under the control of the tcu-1 promoter was performed, and the fold change in cellulase and arabinase activity was calculated. Bars represent the mean ± *SD* of three independent biological replicates. Statistical significance was determined using multiple unpaired *t*-tests (one per row). Asterisks indicate significant differences between the wild type and overexpression strains for each enzyme (*p < 0.05, **p < 0.01, ***p < 0.001, ****p < 0.0001; ns, not significant).

To mine the possibility of developing a cellulase hypersecretory strain, we separately overexpressed lat-1 and xy-31 under the control of the tcu-1 promoter. RT-qPCR analysis was performed to confirm whether the introduced genes were transcriptionally expressed in the respective transformants (Supplementary Figure S4). The results revealed a significant impact of lat-1 overexpression on enzyme activity. Specifically, compared with the control strain, the strain overexpressing lat-1 demonstrated a 1.8-fold increase in cellulase secretion and a 1.5-fold increase in arabinase activity (Figure 7A). The xy-31 overexpressing strain showed a 3-fold and 1.8-fold increase in cellulase and arabinase secretion, respectively (Figure 7B).

These findings suggest that Lat-1 and Xy-31 play crucial roles in regulating the secretion of plant biomass-degrading enzymes. This discovery opens new avenues for developing more efficient microbial strains for industrial applications. Further research could investigate the potential synergistic expression of lat-1 and xy-31 to maximize enzyme production and secretion.

## Discussion

### Arabinose induces the secretion of biomass-degrading enzymes

It is well established that the type of polysaccharide substrate in the growth medium plays a critical role in shaping the diversity of enzymes secreted by these fungi (Wu et al., 2020). However, because polysaccharides are complex, they are not directly involved in regulating cellulase secretion. The regulatory mechanism involves the initial hydrolysis of polysaccharides by membrane-bound enzymes, releasing simple saccharides that act as signals to induce cellulase secretion (Nogueira et al., 2020). The literature indicates that a simple saccharide, arabinose, induces the expression of polysaccharide disintegration genes (Xiong et al., 2004). In another study by Herold et al. (2013), arabinose was shown to induce the xyn1, xyn4, and xyn5 genes in *T. reesei* (Herold et al., 2013). In concordance, we demonstrated that arabinose induces the secretion of hemicellulases and cellulases in *N. crassa* OR74A using biochemical assays, RT-qPCR, and supplementation studies.

### Identification and functional characterization of candidate arabinose transporters

Sugar transporters are the first point of contact for any external stimulus, including inducer saccharides. For example, the role of the Clt-A and Clt-B sugar transporters has been validated in the cellobiose-mediated induction of cellulases in *Aspergillus nidulans* (dos Reis et al., 2016). The study found that deleting the clt-A/B gene significantly reduced cellobiose-mediated induction of cellulases. Similarly, Wang B. et al. (2017) demonstrated that Hgt-1/2, glucose transporters in *N. crassa*, are essential regulators of cellulase transcription and activity (Wang B. et al., 2017). Therefore, we first scrutinized the genome database and identified 40 sugar transporters in *N. crassa*. Based on previously published RNA-seq data, 10 sugar transporter genes were identified as significantly upregulated in response to the polysaccharide substrate, suggesting their potential involvement in polysaccharide-induced expression. Consistent with these transcriptomic observations, RT-qPCR analysis further confirmed the upregulation of all sugar transporter genes under polysaccharide-inducing conditions, implying their key roles in induction.

### Lat-1 and xy-31 are involved in inducing cellulase activity

Lat-1 and Xy-31 are characterized as arabinose transporters (Li et al., 2015; Glass et al., 2014), with Lat-1 identified as a low-affinity arabinose transporter with a Km of 58.12 ± 4.06 mM and a Vmax of 116.7 ± 3.0 mmol/h/g dry cell weight. Although the deletion of the lat-1 gene led to a significantly reduced rate of arabinose utilization, its growth, morphology, and sporulation were largely unaffected. These findings suggest that, while these genes play crucial roles in arabinose metabolism, they are not essential for overall viability or basic cellular functions.

Single-gene knockout experiments revealed a significant reduction in the secretion of biomass-degrading enzymes. This observation suggests that both transporters are essential for the arabinose-mediated induction of saccharification enzymes. The connection between arabinose transport and enzyme production suggests a complex regulatory mechanism in which arabinose transport serves as a signal to trigger the production and secretion of enzymes that break down complex polysaccharides.

Additionally, we observed that the expression of lat-1 and xy-31 is differentially regulated by varying concentrations of extracellular arabinose. Lat-1 was upregulated in a dose-dependent manner, whereas xy-31 was downregulated at lower concentrations. The inverse relationship between lat-1 and xy-31 at low arabinose concentrations may indicate a compensatory mechanism to maintain nutrient balance.

Further to mine the role of transcriptional activators, we also assessed the expression of known transcription factors, such as Xlr-1 and Ara-1, in the deleted strains. Ara-1—classified within the Zn₂Cys₆ binuclear cluster transcription factor family—serves as a key modulator of arabinose metabolism in fungi and yeasts, highlighting its importance in broader regulatory circuits governing carbon source adaptation. Deletion of Ara-1 in *Myceliophthora thermophila* abolishes xylanase and arabinanase secretion on arabinose, suggesting its role in arabinose-mediated induction. Ara-1 specifically binds to conserved cis-elements (e.g., 5’-CCGN₄-₆CCG-3′ or 5’-GGCTAA-3′) in target genes (Gu et al., 2023). Interestingly, these conserved sequences were observed in the promoters of both the lat-1 and xy-31 genes (Data not shown), suggesting a potential role of Ara-1 in Lat-1- and Xy-31-mediated cellulase induction. Similarly, XLR-1 is another important transcriptional activator of hemicellulase gene expression in *Neurospora crassa* (Craig et al., 2015). We analyzed ara-1 and xlr-1 expression and identified significant downregulation in Δlat-1- and Δxy-31-knockout strains, suggesting a feedback loop in which these transporters modulate the regulators of their own expression.

In our efforts to comprehend the combined impact of lat-1 and xy-31 in *N. crassa*, we tried to create a double Δlat-1Δxy-31 knockout strain. Unfortunately, we were unable to obtain any successful clones. The close proximity of lat-1 and xy-31 on the chromosome likely caused crossover interference, which restricted recombination between these genes (Housworth and Stahl, 2003).

### Overexpression of lat-1 and xy-31 in *Neurospora crassa*

Cai et al. (2014) have previously shown that the overexpression of the cellobiose transporter (CDT-2) in *Neurospora* sp. led to a significant increase in cellulase secretion in the fungi (Cai et al., 2014). Therefore, we overexpressed lat-1 and xy-31 in *N. crassa* and assessed the strains for cellulase secretion. The overexpression of lat-1 and xy-31 under the control of the tcu-1 promoter in *N. crassa* has yielded promising results in enhancing the production and secretion of plant biomass-degrading enzymes. The significant increases in cellulase and arabinase activities observed in both overexpression strains highlight the potential of these genetic manipulations for industrial applications. The mechanism by which these transporters increase enzyme secretion warrants further investigation, as understanding this process could lead to more targeted genetic modifications in the future.

## Conclusion

**Figure 8:**
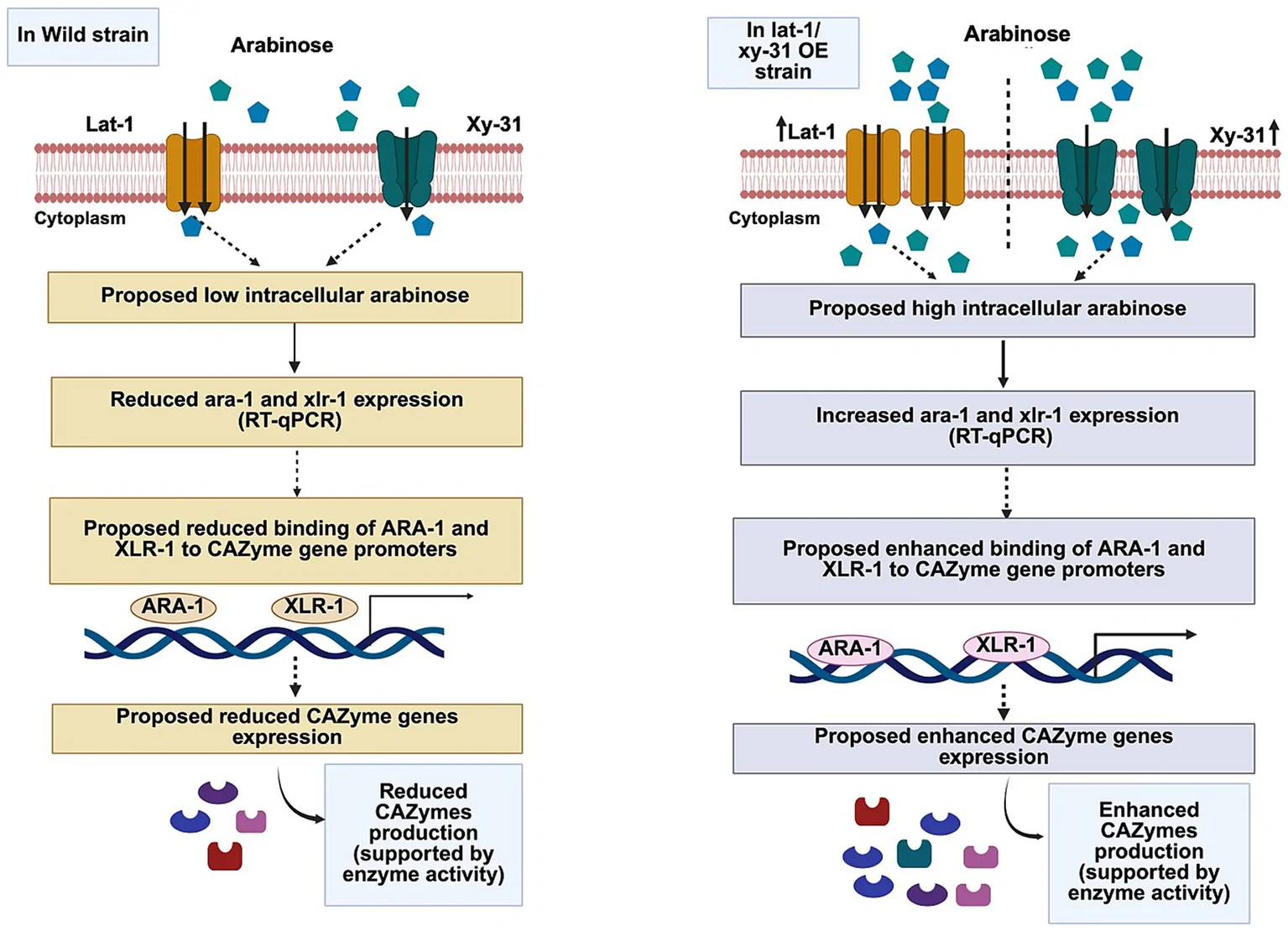
Proposed model summarizing the roles of Lat-1 and Xy-31 in the regulation of arabinose-responsive carbohydrate-active enzyme (CAZyme) production. In the wild-type strain, basal arabinose uptake is possibly mediated by Lat-1 and Xy-31, resulting in basal levels of ara-1 and xlr-1 transcripts. In the lat-1/xy-31 overexpression strain, increased arabinose uptake is proposed to raise intracellular arabinose concentrations, leading to higher ara-1 and xlr-1 transcript levels, as determined by RT-qPCR. The upregulation of these transcription factors is hypothesized to promote the expression of genes encoding CAZymes, thereby increasing CAZyme production, consistent with the elevated enzyme activities observed in this study. This model encapsulates the principal findings of the present study. Solid arrows indicate experimentally supported observations, while dashed arrows represent proposed mechanistic relationships.

This study provides significant insights into the mechanisms of the arabinose-mediated induction of polysaccharide-degrading enzymes in *N. crassa*. Our findings highlight the crucial roles of the arabinose transporters Lat-1 and Xy-31 in this process. These transporters not only facilitate arabinose uptake but also play a key role in signaling the production of cellulase and hemicellulase (Figure 8). The study reveals a complex interplay among these transporters, with their expression and activity finely tuned to extracellular arabinose concentrations. These results enhance our understanding of fungal enzyme regulation and could have important implications for improving industrial enzyme production and biomass degradation strategies.

## Data Availability

All datasets analyzed in this study are accessible through publicly available databases. Transcriptomic datasets and their accession numbers are listed in the manuscript. The reference genome sequence for Neurospora crassa OR74A v2.0 was sourced from the MycoCosm database. Additional data supporting the study's findings are provided within the article and in the Supplementary material.
