# Supplementary material for: Xy-31 and Lat-1 regulate the induction of polysaccharide disintegrating genes by arabinose in Neurospora crassa OR74A
Source: Front Microbiol. 2026 Aug 27;17:1880067. doi: 10.3389/fmicb.2026.1880067 (PMC13558198; doi:10.3389/fmicb.2026.1880067)
Supplement: Supplementary file 1 [file Supplementary_file_1.DOCX]

**Supplementary Information**

**Xy-31 and Lat-1 regulate the induction of polysaccharide disintegrating genes by arabinose in *Neurospora crassa* OR74A**

**Shriya Singh, Nidhi Adlakha***

**Affiliation: Synthetic Biology and Bioprocessing Lab, Regional Centre for Biotechnology, NCR-Biotech Cluster, Faridabad, Haryana-121001**

***Corresponding author: Nidhi Adlakha (**[**nidhi.adlakha@rcb.res.in**](mailto:nidhi.adlakha@rcb.res.in)**)**

**Supplementary Tables**

**Table S1 List of primers for sugar transporters used in RT-qPCR.**

| **PRIMER NAME** | **SEQUENCE** |
| --- | --- |
| NCU02188-FP | CGTGTACCTTGCCGAGAT |
| NCU02188-RP | CACAGCCGTAGCCGATAA |
| NCU05897-FP | CGAGAGAGTGGTGGAAGAG |
| NCU05897-RP | GGAAGAAGAGGATGGTGACA |
| NCU06138-FP | GCACAAGCCGACAATGAG |
| NCU06138-RP | CATAACCAGTATCACTTCCATCAG |
| NCU08152-FP | CCGCCTTCAGAGTTCCTT |
| NCU08152-RP | CGTCCCATCTGTCCTTCTT |
| NCU09287-FP | TGCCGTGGTTCATCAAGA |
| NCU09287-RP | CCTCCTCTGCTCTCCTCT |
| NCU10021-FP | GGTGCTTGGATCTTGATTGG |
| NCU10021-RP | GTGATGACGGCGATGATG |
| NCU00801-FP | ATGTCCTCCGTCTTCTTCC |
| NCU00801-RP | CCGTTGCCGTGATACTTG |
| NCU01132-FP | TGTCGGTGCCATTCTTCA |
| NCU01132-RP | CATCTTGCCTCGCTTGTG |
| NCU05853-FP | ACTGTCTCGCTCCTTGTC |
| NCU05853-RP | ATTCGCCTTGCCTGTCAT |
| NCU00988-FP | GTTACGGCACCTACCTCAT |
| NCU00988-RP | CCGACATCATCTCCACCAT |

**Table S2 List of 40 predicted sugar transporter proteins containing the PF00083 domain identified in the *Neurospora crassa*.**

| **GeneID** | **STs Annotation (FungiDB)** | **ProteinID** | **TMHMM 2.0** | **SignalP** | **WoLF PSORT (Location)** | **STs domain** |
| --- | --- | --- | --- | --- | --- | --- |
| NCU01494 | sut-3 | 3491 | Yes | No | Plasma membrane | PF00083 |
| NCU09287 | sut-18 | 9648 | Yes | No | Plasma membrane | PF00083 |
| NCU01813 | sut-4 | 3199 | Yes | No | Plasma membrane | PF00083 |
| NCU02188 | Lat1 | 10536 | Yes | No | Plasma membrane | PF00083 |
| NCU08152 | sut-6 | 10566 | Yes | No | Plasma membrane | PF00083 |
| NCU05627 | sut-7 | 8885 | Yes | No | Plasma membrane | PF00083 |
| NCU08325 | PHO84 | 5778 | Yes | No | Plasma membrane | PF00083 |
| NCU11342 | sut-24 | 8554 | Yes | No | Plasma membrane | PF00083 |
| NCU05853 | sut-12 | 10733 | Yes | No | Plasma membrane | PF00083 |
| NCU08114 | CDT2 | 84 | Yes | No | Plasma membrane | PF00083 |
| NCU07199 | mfs-27 | 8482 | Yes | No | Plasma membrane | PF00083 |
| NCU04963 | sut-9 | 5420 | Yes | No | Plasma membrane | PF00083 |
| NCU01633 | sut-5 | 3373 | Yes | No | Plasma membrane | PF00083 |
| NCU07169 | sut-23 | 8518 | Yes | No | Plasma membrane | PF00083 |
| NCU08180 | sut-10 | 10599 | Yes | No | Plasma membrane | PF00083 |
| NCU01132 | sut-27 | 7549 | Yes | No | Plasma membrane | PF00083 |
| NCU06522 | sut-2 | 5535 | Yes | No | Plasma membrane | PF00083 |
| NCU07054 | sut-19 | 8651 | Yes | No | Plasma membrane | PF00083 |
| NCU01868 | sut-25 | 588 | Yes | No | Plasma membrane | PF00083 |
| NCU09358 | sut-20 | 3700 | Yes | No | Plasma membrane | PF00083 |
| NCU00988 | mfs-11 | 1693 | Yes | No | Plasma membrane | PF00083 |
| NCU08858 | sut-1 | 6824 | Yes | No | Plasma membrane | PF00083 |
| NCU05597 | ASC3 | 8918 | Yes | No | Plasma membrane | PF00083 |
| NCU05585 | mfs-12 | 8933 | Yes | No | Plasma membrane | PF00083 |
| NCU06846 | mfs-6 | 3053 | Yes | No | Plasma membrane | PF00083 |
| NCU06026 | quinate permease | 10204 | Yes | No | Plasma membrane | PF00083 |
| NCU07861 | hypothetical protein | 3990 | Yes | No | Plasma membrane | PF00083 |
| NCU05350 | hypothetical protein | 2949 | Yes | No | Plasma membrane | PF00083 |
| NCU00801 | CDT-1 | 1907 | Yes | No | Plasma membrane | PF00083 |
| NCU00809 | sut-26 | 1898 | Yes | No | Plasma membrane | PF00083 |
| NCU00821 | sut-15 | 1889 | Yes | No | Plasma membrane | PF00083 |
| NCU06138 | mfs-19 | 10081 | Yes | No | Plasma membrane | PF00083 |
| NCU04537 | sut-22 | 10037 | Yes | No | Plasma membrane | PF00083 |
| NCU06358 | sut-8 | 5896 | Yes | No | Plasma membrane | PF00083 |
| NCU02582 | sor-4 | 763 | Yes | No | Plasma membrane | PF00083 |
| NCU10021 | hgt-1 | 9976 | Yes | No | Plasma membrane | PF00083 |
| NCU06384 | sut-11 | 5864 | Yes | No | Plasma membrane | PF00083 |
| NCU07375 | pho-7 | 1637 | Yes | No | Plasma membrane | PF00083 |
| NCU07607 | SUT 16 | 5243 | Yes | No | Plasma membrane | PF00083 |
| NCU12154 | SUT 29 | 9613 | Yes | No | Plasma membrane | PF00083 |

**Supplementary Figures**

**Fig. S1 Effect of transporter gene deletion on biomass. An equal volume of mycelia was used to inoculate Vogel’s media containing avicel, and a non-significant difference in biomass was observed in lat-1 and xy-31 deleted strains, when compared with the wild-type.**

**Fig. S2 RT-qPCR analysis showing the relative expression of cbh-2 (NCU09680), endoglucanase (NCU00762), endo-β-1,4-xylanase (NCU08189), and arabinosidase (NCU05965) genes in the Δlat-1 and Δxy-31 mutant strains.**

**Fig. S3 RT-PCR was performed to verify the expression of the overexpressed lat-1 and xy-31 genes in each corresponding transformant.**

**Fig. S4 (A) The sequence of selected ten STs was aligned and visualized through NCBI sequence alignment Viewer, suggesting presence of conserved hexose transport domains (PESPR); (B) The sequence of selected ten STs was aligned and visualized through NCBI sequence alignment Viewer, suggesting presence of conserved arabinose transport domains (G-G/FXXX-G).**
